# Force transmission analysis of surface coating materials for multi-fingered robotic grippers

**DOI:** 10.7717/peerj-cs.401

**Published:** 2021-03-18

**Authors:** Gökhan Erdemir

**Affiliations:** Electrical and Electronics Engineering Department, Istanbul Sabahattin Zaim University, İstanbul, Turkey

**Keywords:** Grasp, Force transmission, Material characteristics, Robot gripper, Robot finger coating

## Abstract

Robotic systems are generally used for grasping, carrying, holding, and many similar operations, typically in industrial applications. One of the most important components of robotic systems is robot grippers for the aforementioned operations, which are not only mission-critical but also represent a significant operational cost due to the time and expense associated with replacement. Grasping operations require sensitive and dexterous manipulation ability. As a consequence, tactile materials and sensors are an essential element in effective robot grippers; however, to date, little effort has been invested in the optimization of these systems. This study has set out to develop inexpensive, easily replaced pads, testing two different chemical compositions that are used to produce a tactile material for robot grippers, with the objective of generating cost, time, and environmental savings. Each tactile material produced has its specific individual dimension and weight. First, each of the materials under construction was tested under different constant pressures, and its characteristics were analyzed. Second, each tactile material was mounted on a two-fingered robot gripper and its characteristics. Material characteristics were tested and analyzed as regards their ability to grasp different sizes and types of objects using the two-fingered robot gripper. Based on the analysis of the results the most sensitive and cost-effective material for industrial type multi-fingered grippers was identified.

## Introduction

The design and implementation of robot grippers are one of the essential research topics in robotics, but one where cost-time optimization has thus far not been emphasized. Robot grippers play an important role, especially for industrial applications, where both time and cost are key determining factors. One of the main design criteria for grippers is their ability to grasp and carry an object safely for dexterous and sensitive manipulation (Ma & Dollar, 2017; Spiers et al., 2016; Kumar, Mehta & Chand, 2017; De Oliveira et al., 2015; Morita et al., 2018; Torres-Jara & Natale, 2018; Kappassov, Corrales & Perdereau, 2015; Blanes, Mellado & Beltrán, 2016). The common approach to sense an object with grippers is to use tactile sensors (Kappassov, Corrales & Perdereau, 2015; Dang & Allen, 2014; Girão et al., 2013; Nuelle et al., 2017; Sugaiwa et al., 2010). In the literature, there are numerous different studies that focus on the development of tactile sensors (Kappassov, Corrales & Perdereau, 2015; Birglen & Schlicht, 2017; Natale & Cannata, 2017). There are two common approaches that studies focus on. The first is to embed sensors into coating materials in order to obtain an integrated sensor package or to develop a compound tactile sensor integrated into the coating material (Torres-Jara & Natale, 2018; Guo et al., 2017; Issa et al., 2013). The second approach is to use sensors and coating materials, separately (Spiers et al., 2016; Dang & Allen, 2014; Kim et al., 2006; Abdeetedal & Kermani, 2018). Different types of sensors can be used wrapped with coating materials by taking the second approach (Kim et al., 2006). This method provides certain advantages particularly in terms of decreasing costs (Kumar, Mehta & Chand, 2017; Kappassov, Corrales & Perdereau, 2015; Nuelle et al., 2017; Ulmen & Cutkosky, 2010; Zhang et al., 2020). Another approach to protecting sensory is encapsulation with silicone or similar materials (Hogreve et al., 2020). On the other hand, rapid developments in materials science have played to a significant role in the development of tactile sensors (Shintake et al., 2018). Stretchable electronics components, artificial skins, and thin films are just a few examples (Shintake et al., 2018; Zhu et al., 2018; Wang, Guo & Liu, 2019; Li et al., 2019). Artificial skins are essentially used in humanoid robotics research (Ulmen & Cutkosky, 2010; Silvera-Tawil et al., 2015; Cannata et al., 2008; Saadatzi et al., 2019; Peyre et al., 2019). In some studies in robotics and materials science, artificial skins have been used to sense not only objects but also environmental conditions like temperature, humidity, etc. (Calandra et al., 2015; Kim et al., 2012; Pugach et al., 2016). However, artificial skins are not suitable for industrial applications due to their high cost. A few key criteria come to the forefront when the product life cycle of robotic grippers in industrial applications is examined (Birglen & Schlicht, 2017; Honarpardaz et al., 2017; Kim, Lim & Lee, 2010; Wan & Howe, 2018; Tai et al., 2016; Wolf & Schunk, 2018). These are a long and effective time of operation, low-cost, changeable, or reconfigurable structure, robustness, and stability in sensing (Birglen & Schlicht, 2017; Abdeetedal & Kermani, 2018; Saadatzi et al., 2019; Honarpardaz et al., 2017; Kim, Lim & Lee, 2010; Wan & Howe, 2018; Tai et al., 2016; Wolf & Schunk, 2018). These criteria are important not only for industrial but also for educational and experimental applications where the solution we propose can potentially deliver dramatic cost reductions, prevent waste, and generate meaningful time savings.

This study aims to analyze the force transmissions of two different types of materials with a low-cost pressure sensor (Interlink Electronics, 2010) for robot grippers. Two different material types have been fabricated, namely are epoxy resin-based A and Room Temperature Vulcanization (RTV2) silicone-based B. A low-cost pressure sensor (Interlink Electronics, 2010) was coated with both materials A and B. Subsequently, the force transmission analysis was examined and compared. This article is organized as follows. In the materials and methods section, a detailed description of the two different chemical compounds is presented. An experimental methodology of force transmission analysis is presented at the end of the section. The results of the experimental studies are presented in the results section, under different constant weights and sensing during grasping. Finally, our conclusions are presented in the last section.

## Materials and Methods

In this section, a detailed description is presented for two different chemical compositions with different thicknesses that were produced to coat the surface of the robot fingers. The CAD model designed for the surface coating of the robot gripper fingers was 3D printed to prepare a plaster mold. In the next sub-section, chemical compositions of surface coating materials. Subsequently, the experimental methodology of force transmission analysis for the materials produced is presented in detail.

### Chemical compositions of surface coating materials

Two different material types, A and B, have been prepared using the mold. The details of each material are shown in Tables 1 and 2, respectively. Based on the CAD model, the mold of the materials was produced using gypsum. After casting in the mold, the chemical compositions were kept in a vacuum boiler for 24 h under 60 bars of pressure. Chemical composition, dimensions, and weight values after being removed from the mold are presented in Table 1 for Type A materials, and Table 2 for Type B materials. Both types of materials were chosen, specifically for their different thickness values. The main reason for this is to compare the force transmission characteristics of different materials and the same material sample with different thickness values. All Type A materials composed of epoxy resin and hardener are almost the same weight, width, and length.

**Table 1:** Properties of Type A material.

Material code	Epoxy resin (%)	Epoxy hardener (%)	Width (mm)	Length (mm)	Height/thickness (mm)	Weight (g)
A1	73.22 (~41 g)	26.78 (~15 g)	16	41	1,010	~0.5
A2					1,025	
A3					1,020	
A4					1,240	

**Table 2:** Properties of Type B material.

Material code	RTV2 silicone (%)	RTV2 catalyst (%)	Width (mm)	Length (mm)	Height/thickness (mm)	Weight (g)
B1	96.15 (~50 g)	3.85 (~2 g)	16	41	1,190	~1
B2					1,210	
B3					1,220	
B4					1,260	

All Type B materials composed of RTV2 Silicone and catalyst are almost the same weight, width, and length. Type B materials are heavier and harder than Type A materials because of the density of RTV2 Silicone. The force transmission characteristics of these two different types of materials are compared in detail in the results section.

### An experimental methodology of force transmission analysis

Our experimental methodology of force transmission analysis was planned in two different stages. The first is to analyze the force transmission characteristics of each of the low-cost surface coating materials produced when kept under the constant weight. For this, a 0.2″ diameter force-sensing resistor (FSR) (Interlink Electronics, 2010) was placed on a fixed platform and then covered with the produced materials. Constant weight was applied to the outlined test setup by an increasing load from 10 g to 190 g in 20 g increments. Thus, the force transmission characteristics of each produced material were observed and analyzed under the constant weight.

(1) }{}{V_{\rm out}} = \displaystyle{{{V_i}{R_M}} \over {{R_M} + {R_{\rm FSR}}}}

In Eq. (1), *R*_*M*_ is the measuring resistor (kΩ), *R*_FSR_ is the force-sensing resistor (kΩ), *V*_*i*_ is the input voltage (V), and *V*_out_ is the output voltage (V). The slope angle (α) between *V*_out_ (V) and force (g) is 0.001795 π/rad (Interlink Electronics, 2010). A *V*_*i*_ of +5 V and *R*_*M*_ of 3 kΩ was used. Equation (2) was used to transform *V*_out_ to *F*_measure._ Equation (2) was calculated by using the catalog values of the sensor (Interlink Electronics, 2010).

(2) \displaystyle{{{v_{\rm out}}} \over {{F_{\rm Measure}}}} = 0.001795

The second stage was to analyze the force transmission characteristics during grasping for each of the low-cost surface coating materials produced. To analyze the grasping force, a two-fingered gripper, which is Model T42 (Ma & Dollar, 2017) was built with (Interlink Electronics, 2010). Subsequently, three different kinds of objects were used to measure the force feedback value of the FSR. These were a plastic sphere, an anti-static sponge rectangle prism, and a wood rectangle prism. The condition shown in Eq. (3) was applied to fix the position of the actuator of the gripper. Equations (1) and (2) were again used to transform *V*_out_ to *F*_measure_. In Eq. (3), *F*_*i*_ refers to the measured value of the grasping force (*F*_measure_) at *i* time.

(3) \left( {0.98 \times {F_{i - 1}}} \right) \le {F_i} \le \left( {1.02 \times {F_{i - 1}}} \right)

## Results

### Analysis of sensing under different constant weights

**Figure 1:**
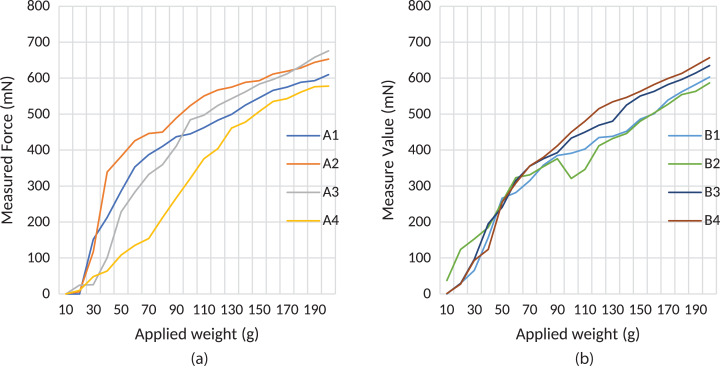
Force transmission characteristics of produced materials under constant weights. (A) Type “A” materials and (B) Type “B” materials.

Materials analysis was performed in two stages. In the first stage, each tactile material's response under constant weight was measured using FSR with 3 KΩ RM resistance. Sensor feedback was gathered in real-time. The sampling time for data acquisition for analysis of sensing under different constant weights was 0.5 s, 100 samples were measured for each weight. The average value of these measurements was then taken as a result. The measured responses of Type A and B materials using FSR are shown in Fig. 1.

According to the test results of the first stage of the study, an almost linear characteristic of the A4 and B4 type tactile materials have been observed. The sample A4 has greater linear force transmission characteristics than other Types A samples, as seen in Fig. 1A. It can be said that the main reason for this is that due to their structural properties, materials produced from epoxy resin become more stable as the material thickness increases. However, although its characteristics are almost linear, sensitivity decreases as thickness increases.

The same behavior is observed in the Type B samples. Sample B3 is thicker than the other samples, and its linear force transmission characteristics are more linear than the other Type B samples, as shown in Fig. 1B. Based on the results of our tests, the most suitable thickness for both Type A and B materials is 1.25–1.30 mm. In Fig. 1B, the unexpected response of sample B2 is seen when a weight between 100 and 120 g was applied. Experiments were repeated with different FSRs to explain the reason for this response. This range may be a hysteresis zone for FSR. However, the response can be considered linear since the changes in this range are small. Different kinds of filters can be used to compensate for this effect and also linearization can be applied. The aim of this study is to share raw results with researchers. According to different types of applications, researchers can choose the most effective approach for their particular research. Usage of the materials with suitable control such as in Huynh & Kuo (2020) can linearize the hysteresis part of the B2 material. As seen in Fig. 1, when the thickness of the Type A materials is increased, the transmitted force value decreases due to the material’s soft structure. Conversely, it is observed that the transmitted force value increases when the thickness of the Type B materials is increased.

### Analysis of sensing during grasping

**Figure 2:**
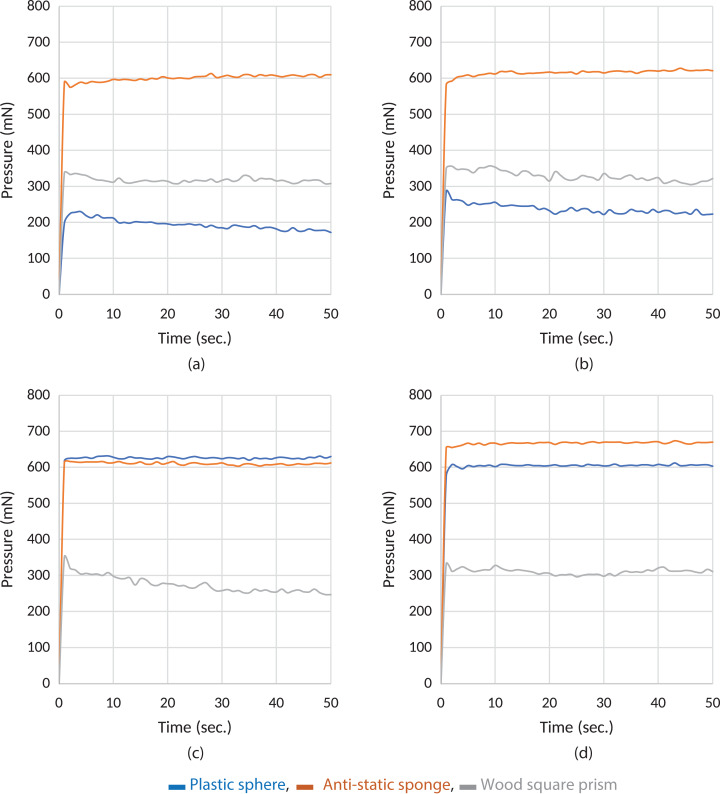
Experiment results of grasping of different items with the Type A material. (A) A1, (B) A2, (C) A3 and (D) A4, respectively.

**Figure 3:**
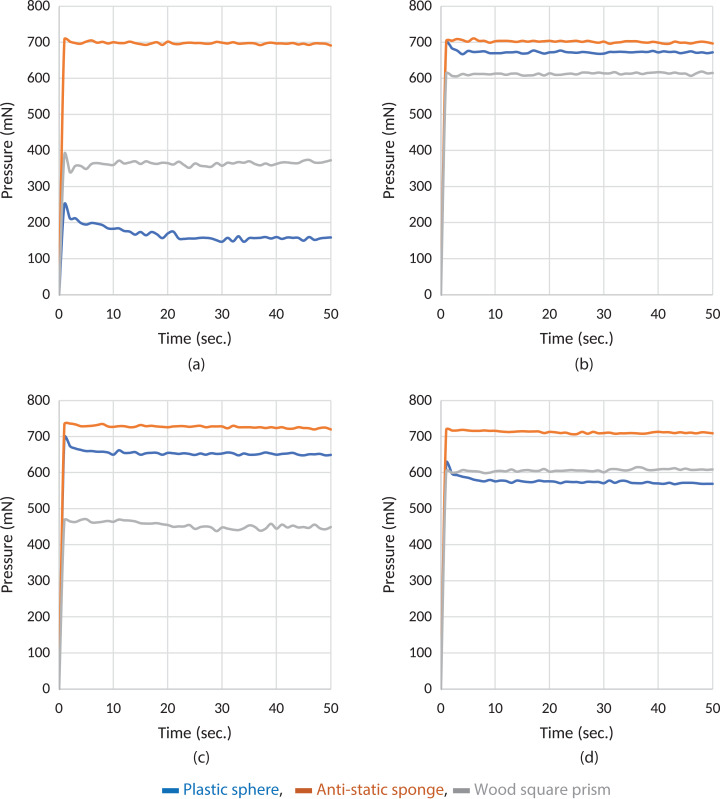
Experiment results of grasping of different items with the Type B material. (A) B1, (B) B2, (C) B3 and (D) B4, respectively.

The second part of the experimental studies analyzed the force transmission characteristics of the materials during the grasp operation of a multi-fingered robot gripper manipulating three different kinds of objects, as shown in Table 3. Model T42 gripper (Ma & Dollar, 2017) was used for the experimental grasping studies. The sampling time for this considered for the experimental case was 1 s. Measurement results have been presented as a Supplemental File. The test results of Type A and B materials are shown in Figs. 2 and 3, respectively. The correlation of Eq. (3) was used to lock the robot gripper's actuator as a controller to all experiments in this stage. A plastic sphere, an anti-static sponge rectangle prism, and a wood rectangle prism were used as grasped objects in this experiment, details of which are presented in Table 3.

**Table 3:** Properties of items which are used for grasping analysis.

Material	Shape	Dimensions (mm)	Image
Plastic	Sphere	Diameter: 73.25	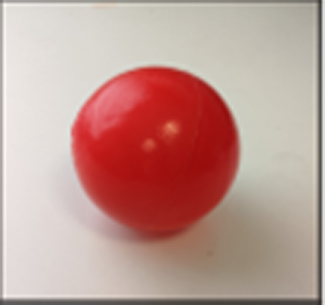
Anti-static sponge	Rectangle prism	Length: 49.50	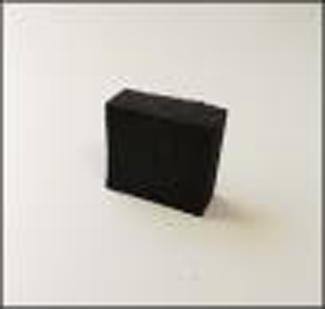
		Height: 19.40	
		Width: 19.40	
Wood	Rectangle prism	Length: 29.00	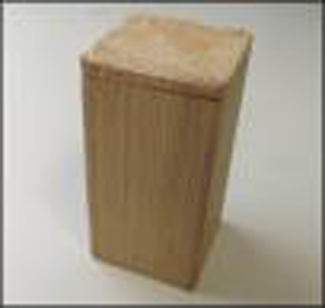
		Height: 116.08	
		Width: 29.0	

As seen in Fig. 2D, sample A4 produces a more stable response during grasping operations with all three objects. In all the experimental graphs shown in Fig. 2, it is observed that there is a ripple in the response of a wood rectangle prism. This is because the wood rectangle prism slips down, as it is heavier than other materials when the correlation in Eq. (3) is applied. The controller runs the actuator again using the correlation in Eq. (3) to simultaneously stabilize slipping. According to the test results, it can be determined that the controller produces a stable response. In short, it can be seen that sample A4 produces more stable and reliable force transmission characteristics in Fig. 2.

As seen in Fig. 3D, the samples B2 and B4 produce a more stable response during grasping operation for all three objects as does A4. There are ripples in the wood rectangle prism graphics again for the reason explained previously. It can be said that the controller also produces a satisfactory response to tolerate slipping in this stage. Samples B2 and B4 produce a more stable and reliable force transmission characteristic in Fig. 3.

## Discussion

Two different epoxy-based and RTV2 silicone-based material types have been compared and analyzed to determine both materials’ force transmission capabilities. Based on the test results, it is clear that Type A and Type B materials can be used effectively in low-cost industrial robot gripper applications. It has also been demonstrated in the experimental results that these materials transmit grasping force to sensors almost linearly. In the common approach, robot finger coating materials are designed as a fixed part of robot fingers. However, this approach makes maintenance, and finger/coating changes costly. Using the materials tested in this study, it will be possible to achieve a serious cost reduction during maintenance operations on robot grippers as instead of discarding the entire finger as is currently done, and it would be possible simply to replace a very thin and inexpensive coating. Consequently, this research will also assert new and innovative approaches to robot finger design that take this approach to reduce cost and waste without sacrificing, and possibly even enhancing, performance.

## Conclusion

In this study, two different chemical compounds were used to produce low-cost surface coating materials for multi-fingered robot grippers. The two different material Type A and Type B, or epoxy-based and RTV2 silicone-based, have been composed to cover the FSR of the robot finger. The details of each material were presented in Tables 1 and 2, respectively. Two different experimental setups were designed in order to conduct force transmission analyses. The first setup was designed to analyze the force transmission under the constant weight. The second was designed in order to observe characteristic behavior during grasping operations. According to the test results, it is clearly demonstrated that both Type A and Type B materials, as well as FSR, can be used in low-cost industrial applications, where it has been demonstrated that they deliver clear benefits in terms of cost, time, and environmental impact over the current procedure of disposing of the entire finger by allowing for the replacement of only a small, inexpensive coating pad, which can be changed much more quickly and by less-skilled technicians than an entire finger. However, the correlation established in Eq. (2) should be considered when determining material thickness. The test results show that the A4 and B4 samples display almost linear force transmission characteristics for both experiments. The results underlined that the most suitable thickness for both Type A and Type B materials is 1.25–1.30. When deployed for industrial applications, coatings made of either material should be replaced monthly, because it has been determined that the structural properties of both materials are integrated under prolonged use. In future work, studies will be made of these two types of materials when applied to multi-fingered robot grippers, such as three-fingers, four-fingers structures in order to verify that the materials deliver similar benefits in these more challenging gripping environments.

## Supplemental Information

10.7717/peerj-cs.401/supp-1 Supplemental Information 1 Experimental test results.

## Additional Information and Declarations

### Data Availability

The following information was supplied regarding data availability: Experimental test results are available as a Supplemental File.
